# Resveratrol Inhibits Alpha-Melanocyte-Stimulating Hormone Signaling, Viability, and Invasiveness in Melanoma Cells

**DOI:** 10.1155/2013/632121

**Published:** 2013-05-23

**Authors:** Yu-Jen Chen, Ying-Yin Chen, Yi-Feng Lin, Hsuan-Yun Hu, Hui-Fen Liao

**Affiliations:** ^1^Department of Radiation Oncology, Mackay Memorial Hospital, Taipei 104, Taiwan; ^2^Institute of Traditional Medicine, National Yang Ming University, Taipei 112, Taiwan; ^3^Department & Graduate Institute of Pharmacology, College of Medicine, Taipei Medical University, Taipei 110, Taiwan; ^4^Department of Biochemical Science and Technology, National Chiayi University, 300 University Road, Chiayi 600, Taiwan

## Abstract

Melanoma is a malignancy with high potential to invasion and treatment resistance. The **α**-melanocyte-stimulating hormone (**α**-MSH) signal transduction involving Wnt/**β**-catenin, c-Kit, and microphthalmia-associated transcription factor (MITF), a known pathway to produce melanin, has been demonstrated as one of cancer stem cell characteristics. This study was aimed to examine the effect of resveratrol, an abundant ingredient of grape and medicinal plants, on **α**-MSH signaling, viability, and invasiveness in melanoma cells. By **α**-MSH treatment, the melanin production in B16 melanoma cells was augmented as a validation for activation of **α**-MSH signaling. The upregulated expression of **α**-MSH signaling-related molecules **β**-catenin, c-Kit, and MITF was suppressed by resveratrol and/or STI571 treatment. Nuclear translocation of MITF, a hallmark of **α**-MSH signaling activation, was inhibited by combined treatment of resveratrol and STI571. At effective concentration, resveratrol and/or STI571 inhibited cell viability and **α**-MSH-activated matrix metalloproteinase- (MMP-)9 expression and invasion capacity of B16 melanoma cells. In conclusion, resveratrol enhances STI571 effect on suppressing the **α**-MSH signaling, viability, and invasiveness in melanoma cells. It implicates that resveratrol may have potential to modulate the cancer stem cell characteristics of melanoma.

## 1. Introduction 

Melanoma is the most frequent cause of mortality in skin cancer with highly potential for widespread metastasis [1]. The UV-induced damage having distinct mutational signatures including C to T transitions may increase the risk of melanoma [2]. The *α*-melanocyte-stimulating hormone (*α*-MSH) is required for the development of melanin in human skin and hair [3, 4]. *α*-MSH binds to its specific receptor (MC1R) and increases cAMP, which activates melanogenesis by activating a melanocyte specific transcription factor, microphthalmia-associated transcription factor (MITF) [5]. MITF is a transcription factor contained in several cell types and is involved in the regulation of melanocyte differentiation, pigmentation, proliferation, and survival of cells [6]. 

Isoform MITF-M acts as dual roles in the Wnt signaling pathway and functions as both a nuclear target and a nuclear mediator of Wnt/*β*-catenin signaling [7]. The binding of Wnt to its receptor Frizzled leads to inactivation of glycogen synthase kinase-3*β*, followed by the accumulation of *β*-catenin and its translocation to nucleus. Wnt/*β*-catenin signaling upregulates MITF-M expression through the interaction with a member of the lymphoid enhancing factor-1 (LEF-1) [7]. 

c-Kit (CD117), a receptor tyrosine kinase of stem cell factor (SCF), is a proto-oncogene that can serve as a potential target for molecular therapy of metastatic melanoma [8]. The expression of c-Kit in the majority of mucosal melanomas suggests that it may be useful in the assessment of these tumors for potential treatment with tyrosine kinase inhibitors, such as Imatinib [9]. Imatinib (STI571), a selective inhibitor targeting Abl as well as c-Kit and the platelet-derived growth factor receptor, has been tested for the efficacy and toxicities in metastatic melanoma patients, suggesting that c-Kit might be a drugging target for treatment of melanoma [8]. 

Cancer stem cells (CSCs) or cancer initiating cells are a subset of tumor cells able to show self-renewal and differentiation. CSCs, which express stemness properties, are hypothesized to be responsible for tumorigenesis, metastasis, and resistance to cancer treatment [10]. Growing number of research groups isolated and identified CSCs, by appropriate selection markers, in various malignancies, such as leukemia, melanoma, breast, brain, colon, pancreas, and liver cancers [11]. In melanoma, CSCs tend to express developmental genes and stem cell markers including Notch receptors, Wnt proteins, CD133, c-Kit/CD117, and nestin antigens [12]. Recent articles have described that CD133^+^ melanoma stem cells expressed certain ABC transporters to efflux drugs, such as ABCB5 protein, and may cause the chemotherapeutic resistance [13]. Additionally, MITF, regulating melanocytes and melanoma development, has also been reported as a factor supporting melanoma stem cell properties [14, 15]. 

Conventional cytotoxic agents are designed to eliminate proliferation of cancer cells. However, this strategy is not sufficient to inhibit the CSCs and, by contrast, may increase tumor relapse, metastasis, and resistance to further chemotherapy. Complementary and alternative medicine (CAM) has been demonstrated that may be effective in CSC inhibition. For examples, salinomycin inhibited the proliferation, migration, and invasion of human endometrial cancer stem-like cells [16]. Resveratrol, a natural polyphenolic polyphenol isolated from red wine and traditional Chinese medicine *Polygonum cuspidatum*, possesses several biological activities including antitumor, anti-inflammation, and antiaging effects [17]. Resveratrol combined with conventional chemotherapy treatment eliminated tumor-initiating stem-like and epithelial-mesenchymal transition (EMT) properties in malignant head and neck cancers [17]. However, the effect and possible pathways of resveratrol in regulating growth of melanoma are still unclear. 

The present study are aimed to further examine the role of *α*-melanocyte-stimulating hormone (*α*-MSH) signal transduction involving Wnt/*β*-catenin, c-Kit, and MITF, known pathways to produce melanin and have been considered as CSC-associated markers in melanoma. The effects of resveratrol, an abundant ingredient of foods and medicinal plants, with and without STI571 (imatinib mesylate) treatment on *α*-MSH signaling, viability, and invasiveness in melanoma cells were examined. 

## 2. Materials and Methods 

### 2.1. Materials and Cells

 STI571 (also named imatinib mesylate) was kindly provided from Novartis Pharmaceutical Co. (Basel, Switzerland). Resveratrol, *α*-MSH, and reagents used in this study were purchased from Sigma-Aldrich (St. Louis, MO, USA). Murine melanoma B16 cells were purchased from the American Type Culture Collection (ATCC, Manassas, VA, USA) and were maintained in medium containing Dulbecco's modified Eagle's medium (DMEM; Gibco, Invitrogen Corporation, NY, USA) supplemented with 10% fetal bovine serum (Gibco, Grand Island, NY, USA) and 2 mM L-glutamine (Sigma) and incubated in an incubator (5% CO_2_, 37°C).

### 2.2. Melanin Content Assay

 The treated cells were collected, washed with phosphate-buffered saline (PBS, 137 mM NaCl, 12 mM Phosphate, 2.7 mM KCl, pH 7.4), and centrifuged at 12,000 rpm for 10 min. The supernatant was removed and the pellet was added with NaOH (1 M) and reacted at 60°C for 3 h. Then, the aliquots of cell lysates were placed in 96-well plates, and the amount of melanin was determined by measuring the absorbance at 400 nm using an enzyme-linked immune-sorbent assay (ELISA) reader. Melanin contents were expressed as percentages of those of untreated controls. 

### 2.3. Western Blot Analysis

The treated cells were collected, lysed, and isolated the total proteins. Protein samples were quantified using a bicinchoninic acid (BCA) protein assay kit (Bio-Rad, Hercules, CA, USA), and the same amount of protein (100 *μ*g per well) was disrupted with 2x concentrated electrophoresis sample buffer (1 M Tris, pH 6.8, 5% SDS, 40% glycerol, 0.005% bromophenol blue, and 8%  *β*-mercaptoethanol). After analyzing the samples by subjecting to 10% sodium dodecyl sulfate (SDS) polyacrylamide gel electrophoresis, the protein gel was transferred to a polyvinylidene difluoride (PVDF) membrane, blotted with primary antibodies, including anti-Wnt-1 (1 : 200 dilution, BioSource, Bethesda, MD, USA), anti-*β*-catenin (1 : 200 dilution, Santa Cruz Biotechnology, Dallas, TX, USA), anti-MITF (1 : 200 dilution, Santa Cruz), anti-MMP-9 (1 : 1000 dilution, Santa Cruz), anti-histone H3 (1 : 500 dilution, Epitomic, Burlingame, CA, USA), and anti-glyceraldehyde-3-phosphate dehydrogenase (GAPDH, 1 : 1000 dilution, Santa Cruz), and then incubated at 4°C for overnight. The membrane was further hybridized with horseradish peroxidase (HRP) conjugated secondary antibody (1 : 1000 dilution, Santa Cruz) for 1.5 h followed by exposing with enhanced chemiluminescence (Perkin Elmer, Waltham, MA, USA). Relative protein levels were determined by densitometry using ImageJ software (Version 1.36b, NIH, Bethesda, MD, USA) with normalization to the internal control. 

### 2.4. Immunofluorescence Staining of c-Kit

 The treated cells were collected, washed with PBS (contain 5% Bovine serum albumin, BSA), and reacted with anti-c-Kit/CD117/SCFR primary antibody (1 : 200 dilution, Bioss Inc., Woburn, MA, USA) and immunofluorescence FITC-conjugated anti-IgG-TR antibody (1 : 500 dilution, Bioss Inc.) in order to determine the distribution of c-Kit in cells. The percentage of c-Kit-positive cells was analyzed by a NucleoCounter NC-3000 system (ChemoMetec A/S, Allerød, Denmark), and the cell morphology was then photographed under a fluorescence microscope at a magnification of 200x. 

### 2.5. Preparation of Nuclear and Cytosolic Extracts from Cells

To prepare the cytosolic and nuclear proteins, the nuclear extraction buffer (500 mM NaCl, 1.5 mM MgCl_2_, 0.2 mM EDTA, 1 mM DTT, 20% Glycerol, 0.1% Triton X-100, and 20 mM HEPES, pH 7.4) was used to separate the cell nuclei from the cytosolic extract. Then, the nuclei were resuspended in lysis buffer (150 mM NaCl, 0.1% SDS, 1% Triton X-100, and 100 mM Tris, pH 8.0) and centrifuged at 400 g for 10 min. The nuclear proteins were collected and stored at −80°C. The protein concentrations were determined using a BCA protein assay kit, and the expression of cytosolic and nuclear MITF was assayed by Western blotting. 

### 2.6. Cell Viability Assay

 B16 melanoma cells (2 × 10^5^ cells/mL) were cultured for 12 h and then incubated with *α*-MSH (10 nM), STI571 (20 *μ*M), and/or Resveratrol (15 *μ*M) for a further 72 h. Then, the treated cells were collected and cell viability was measured by trypan blue dye exclusion test and observed under a microscope at a magnification of 100x.

### 2.7. Cell Invasion Assay

 Assays of cell invasion properties were performed using a modified Boyden chamber with polyethylene terephthalate filter inserts coated with a Matrigel matrix (BD Biosciences, NJ, USA) in 24-well plates containing 8 mm pores. In brief, 10^5^ cells were suspended in a serum-free medium with 0.5% BSA. Then, cells were plated into the upper chamber followed by filling the lower chamber with the same medium with or without *α*-MSH (10 nM), STI571 (20 *μ*M), and/or resveratrol (15 *μ*M). Cells were incubated for 24 h, the noninvading cells were gently removed, and the cells invading the lower side were stained with Liu's stain (Sigma) and counted by microscopic examination. 

### 2.8. Statistical Analysis

Data were obtained from three independent experiments and expressed as the mean ± standard error (SE). Statistical comparisons were based on Student's *t*-test or analysis of variance. Differences were considered significant at *P* < 0.05. All statistical analyses were carried out using SigmaStat and Sigma Plot software (Jandel Scientific, San Rafael, CA). 

## 3. Results 

### 3.1. Effect of *α*-MSH on Melanoma B16 Cells

**Figure 1:**
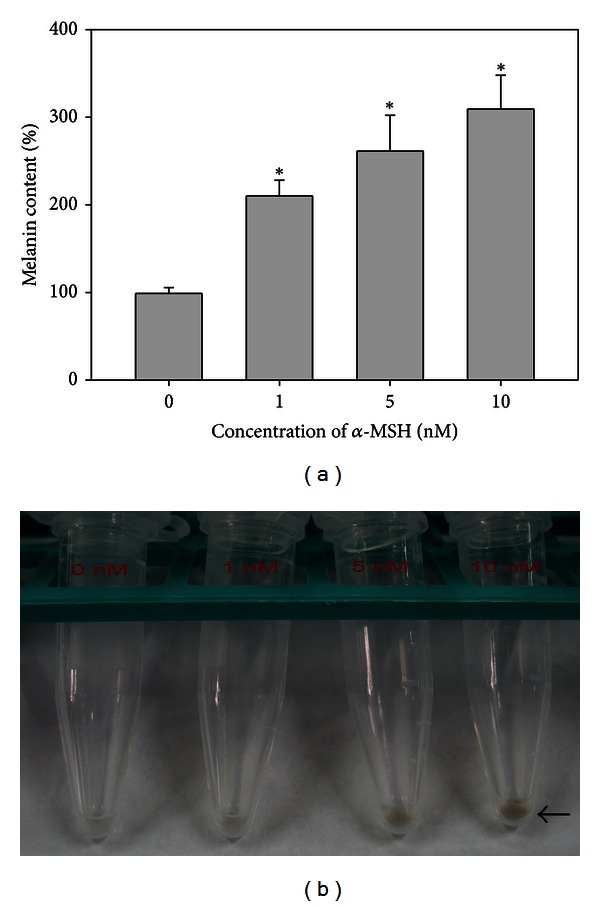
Effect of *α*-MSH on melanin synthesis in melanoma B16 cells. (a) Melanin content in *α*-MSH (0–10 nM) treated cells. (b) Melanin expression in the treated cell pellet. *P < 0.05 as compared to the control group. The results were calculated from three independent experiments and expressed as mean ± standard error (SE).

*α*-MSH stimulated the melanin expression in B16 cells in a concentration-dependent manner. In Figure 1(a), B16 cells treated with *α*-MSH (0–10 nM) markedly increased the melanin content. In Figure 1(b), melanin was determined by measuring the absorbance at 400 nm using an ELISA reader. We further tested the signaling molecule expression, cells viability, and invasiveness in melanoma cells with and without the treatment of 10 nM *α*-MSH. 

### 3.2. Expression of Wnt/*β*-Catenin in B16 Cells

**Figure 2:**
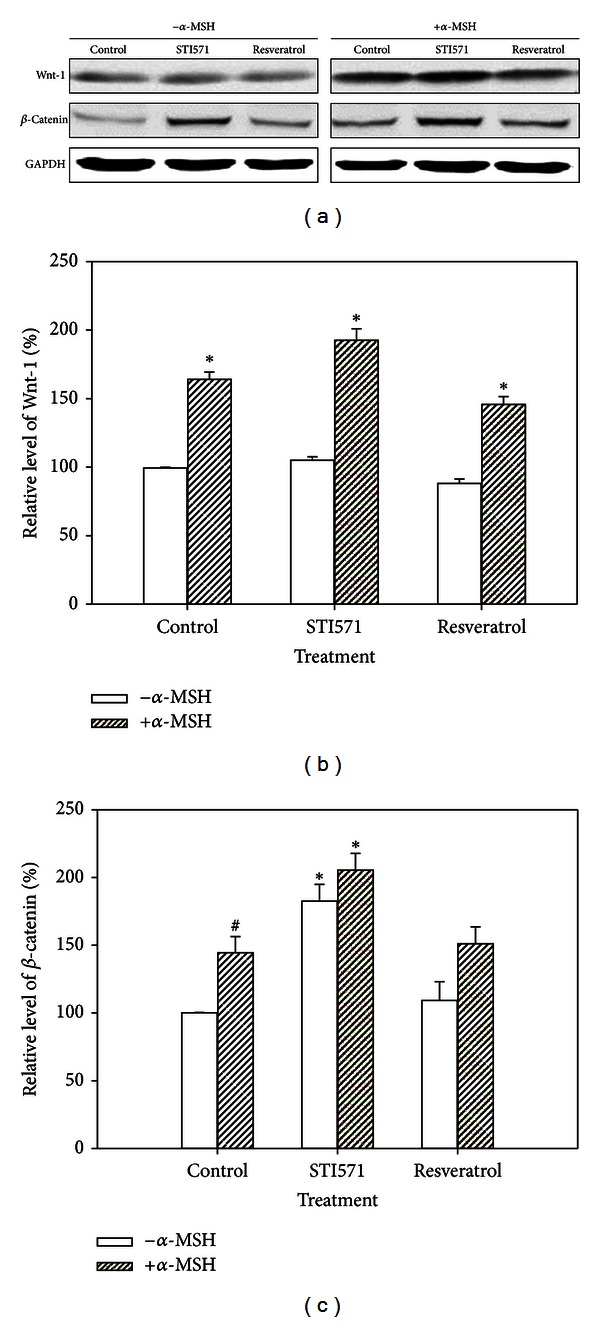
Expression of Wnt-1 and *β*-catenin in B16 cells with *α*-MSH (10 nM), STI571 (20 *μ*M), and/or resveratrol (15 *μ*M) treatment by using the Western blot analysis. The relative values were normalized to the internal control GAPDH and the data were expressed as mean ± SE. *P < 0.05 as compared to the control group. ^#^ *P* < 0.05 as compared between *α*-MSH treatment and untreated group.

*α*-MSH upregulated Wnt-1 and *β*-catenin expression in B16 cells. In Figure 2, the expression of Wnt-1 was not changed when the cells treated with STI571 or resveratrol. STI571 treatment significantly increased the *β*-catenin level in cells, but resveratrol did not have such effect. 

### 3.3. Expression of c-Kit in B16 Cells

**Figure 3:**
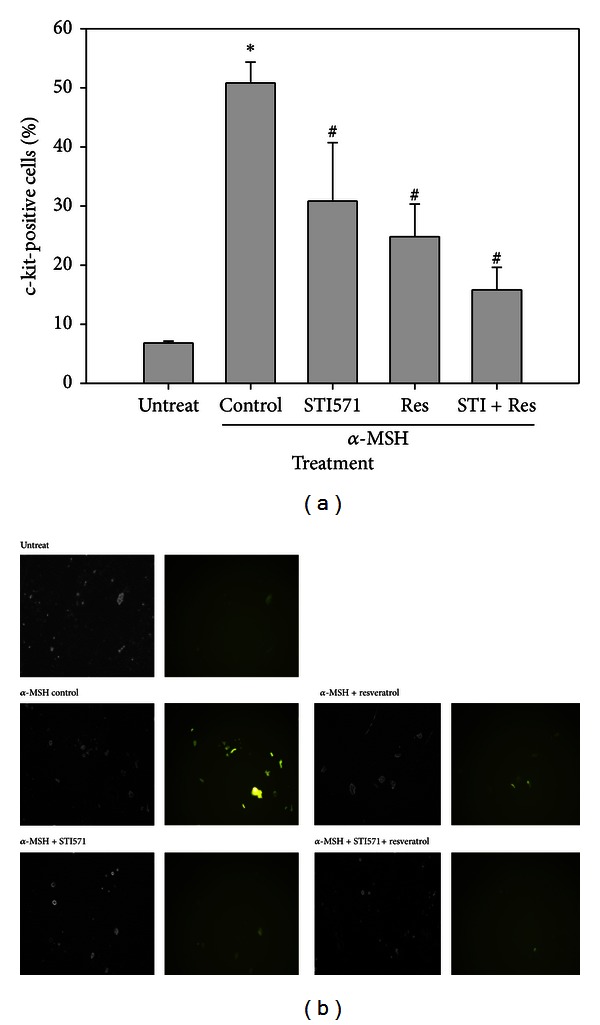
Expression of c-Kit on cell surface. (a) Percentage of c-Kit-positive cells. (b) Immunofluorescence staining of c-Kit. These cells were stained with FITC-conjugated anti-c-Kit antibody (green) and observed under a microscope (200x). *P < 0.05 as compared to the untreated group. ^#^ *P* < 0.05 as compared to the *α*-MSH alone group. Res: resveratrol; STI: STI571.

 By immunofluorescence staining, the expression of surface marker c-Kit was up-regulated by *α*-MSH treatment in B16 cells, with both the percent of c-Kit-positive cells (Figure 3(a)) and fluorescence intensity (Figure 3(b)). STI571 and/or resveratrol significantly decreased the c-Kit level that increased by *α*-MSH (Figures 3(a) and 3(b)). 

### 3.4. Expression of Cytosolic and Nuclear MITF in B16 Cells

**Figure 4:**
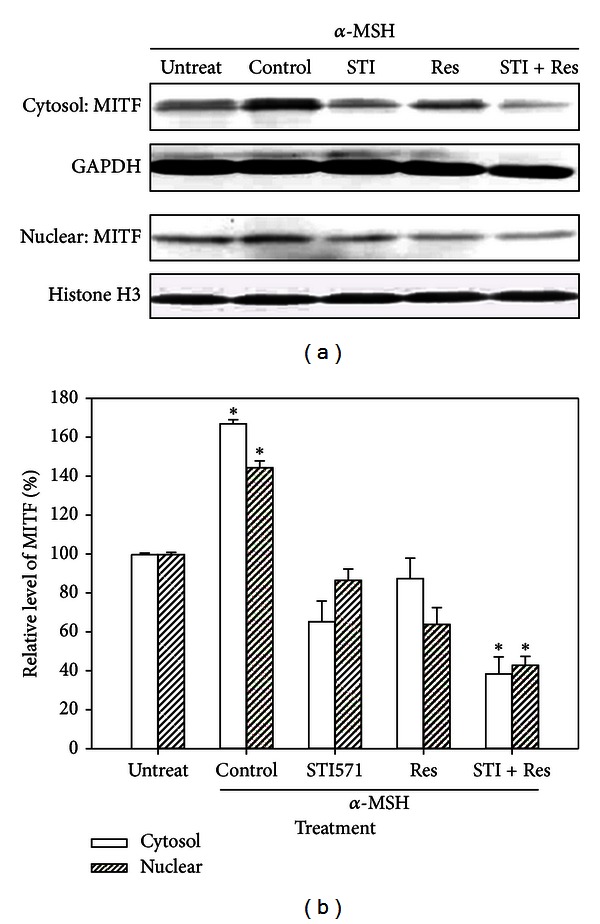
Expression of nuclear and cytosolic MITF in B16 cells with *α*-MSH (10 nM), STI571 (20 *μ*M), and/or resveratrol (15 *μ*M) treatment by using the Western blot analysis. The relative values normalized to the internal control GAPDH (cytosol) and histone H3 (nuclear) were calculated from three independent experiments. Data were expressed as mean ± SE. *P < 0.05 as compared to the control group. Res: resveratrol; STI: STI571.

 In Figure 4, the cytosolic and nuclear proteins were isolated from the treated cells and assayed the expression of MITF. *α*-MSH-treated B16 cells significantly increased MITF expression in both cytosolic and nuclear fractions. STI571 combined treatment with resveratrol markedly decreased both cytosolic and nuclear MITF levels that increased by *α*-MSH. 

### 3.5. Effect of Resveratrol on *α*-MSH Signaling, Viability, and Invasiveness

**Figure 5:**
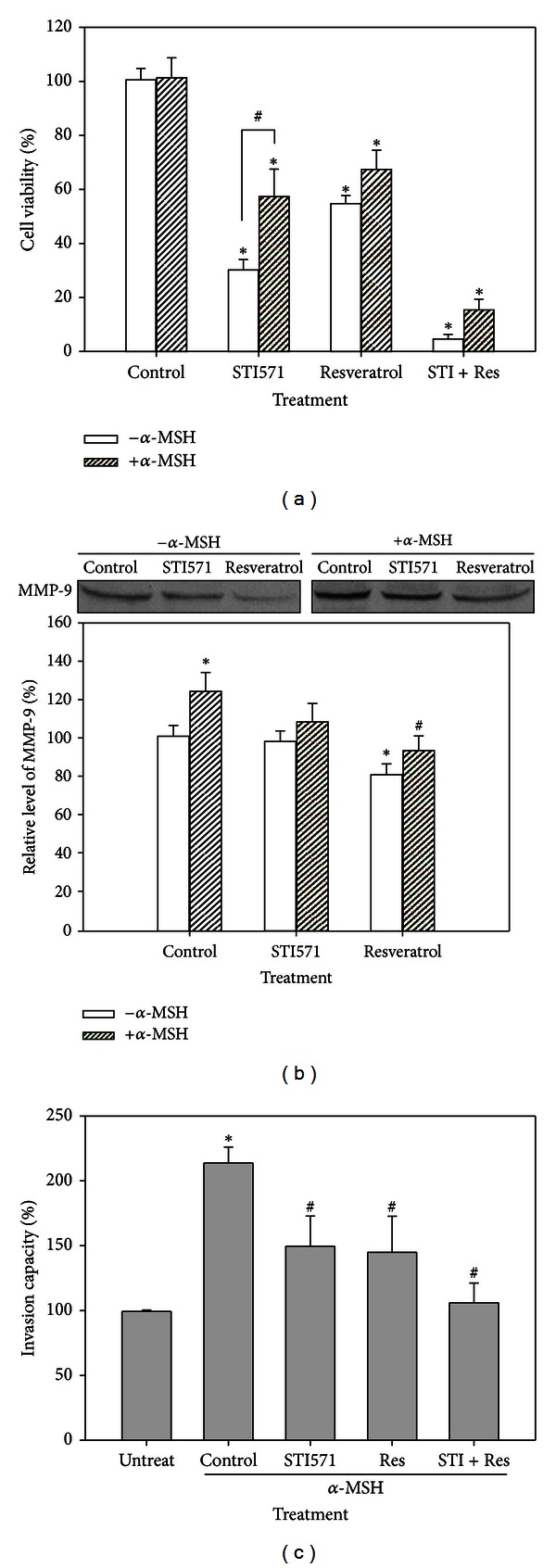
Effect of resveratrol on cell viability, MMP-9 expression, and invasion capacity in B16 cells with *α*-MSH (10 nM), STI571 (20 *μ*M), and/or resveratrol (15 *μ*M) treatment. (a) Viability assay by using a trypan blue dye exclusion test. (b) MMP-9 expression by Western blotting. (c) Cell invasion assay. *P < 0.05 as compared to the control group. ^#^ *P* < 0.05 as compared between *α*-MSH treatment and untreated group. Res: resveratrol; STI: STI571.

 In Figure 5(a), resveratrol and combined treatment with STI571 significantly decreased the viability of B16 cells with and without *α*-MSH treatment. In Figure 5(b), *α*-MSH treatment increased MMP-9 expression in B16 cells. Resveratrol, but not STI571, significantly decreased MMP-9 level in cells with and without *α*-MSH treatment. Additionally, B16 cells with *α*-MSH treatment markedly increased invasion capacity. As shown in Figure 5(c), resveratrol and combined treatment with STI571 significantly inhibited the invasion of cells stimulated by *α*-MSH.

## 4. Discussion 

The incidence of melanoma has been rising at an alarming rate in both men and women causing mortality and resistance to current therapies [18]. The present study stimulated melanoma B16 cells with *α*-MSH and demonstrated the increased expression of melanin production to be accompanied with the upregulation of CSC-associated molecules (Wnt-1/*β*-catenin, c-Kit, MITF, and MMP-9) and invasion ability. Resveratrol, a natural product isolated from traditional Chinese medicine (*Rheum officinale* Baill. and *Polygonum cuspidatum*) and foods (grape skin, red wine, cranberries, blueberries, and peanuts) [19, 20], alone or combined treatment with drug STI571 was effective in inhibiting the abovementioned molecule expression, decreasing the cell viability, as well as suppressing the invasion of melanoma B16 cells. For further development of a new treatment strategy in the future, the pharmacological kinetic profiles of oral administration of resveratrol and STI571 are necessary to clarify the possible mechanism of combination *in vivo*. 

Epidermal keratinocytes and melanocytes have been the subject of many skin biology studies because they respond to a rich variety of inflammatory and immunomodulating cytokines, hormones, vitamins, UV light, toxins, and physical injury [21]. Melanin is produced in melanocytes and melanomas through metabolism of melanogenic enzymes, such as tyrosinase. Certain pathways, including *α*-MSH, Wnt/*β*-catenin, c-Kit, and their downstream modulation of MITF signaling, receive signals from receptors and initiate melanogenesis process [22]. Articles reported that resveratrol exhibited the inhibitory activity against tyrosinase and MITF may have potential in melanogenesis inhibition [23, 24]. This study treated melanoma B16 cells with *α*-MSH and demonstrated that the melanin level was increased in a concentration-dependent manner (Figure 1). The *α*-MSH-mediated activation also stimulated Wnt/*β*-catenin and c-Kit up-regulation, an experimental model resembling clinical melanoma development. In embryonic and adult cells, the Wnt/*β*-catenin pathway involved several cellular activities, such as cell proliferation, migration, and differentiation [25]. *β*-catenin, an important intermediate in Wnt signaling pathway, has been identified as a key point for melanocyte development [26]. c-Kit (CD117), the receptor for the stem cell factor (SCFR), is a growth factor for melanocyte migration and proliferation and has been shown differential expression in various malignant melanocytic lesions with dermis invasion and to differentiate metastatic melanoma from primary melanoma [27]. Additionally, *α*-MSH is a physiological ligand that binds to melanocortin-1 receptor, initiates signal transduction to induce transcription factor MITF expression, and then leads to increase in melanin synthesis [5]. 

Among skin cancers, melanoma responds poorly to chemotherapy. For examples, melanoma B16/PDGF-BB cells have reported not being sensitive to paclitaxel, but that combination of tyrosine kinase inhibitors (such as imatinib and vatalanib) could increase the inhibitory effects, suggesting a novel target for the treatment of melanomas expressing c-Kit [28]. MITF and P27 are the key molecules that switch the transition between melanoma-initiating cells and their differentiated progeny. Therefore, the CDK inhibitor P27 is increased in MITF-depleted cells and is required for exacerbation of the tumorigenic properties of melanoma cells [29]. Like CSCs, the expression of melanogenic molecules, such as Wnt/*β*-catenin, c-Kit, and MITF, in melanoma exhibits strong morphological, functional, and molecular heterogeneity that might reflect the existence of different cancer cell populations. In melanoma B16 cells, the present study demonstrated that the CSC-associated molecules Wnt/*β*-catenin, c-Kit, and MITF were up-regulated by the stimulation of *α*-MSH. The expression of MMP-9 and the invasion capacity were also increased in *α*-MSH-treated B16 cell. It was suggested that *α*-MSH might induced the melanoma cell populations toward stem-like properties, causing the cells to be more resistant to chemotherapy and more prone to metastasis. 

Resveratrol, a phytochemical widely found in foods and in traditional Chinese medicines, has been reported that possesses various bioactivities in cancer cells [30–32]. For examples, resveratrol prevents injury of endothelial cells in high-dose interleukin-2 therapy against melanoma [30]. In chronic myeloid leukemic K562 cells, resveratrol acts as a Bcr-Abl inhibitor and suppresses Sonic hedgehog (Shh) signaling, another CSC signaling pathway, in both STI571-sensitive and -resistant cells [31]. Resveratrol also reduces IL-6-mediated Shh signal expression in acute myeloid leukemia [32]. Although articles reported that resveratrol inhibits tumor-initiating stem-like cells properties in head and neck cancer [17], breast cancer [33], glioblastoma [34], and pancreatic cancer [35], there is no research indicating the effect of resveratrol on CSCs of melanoma. In this study, we first demonstrated that resveratrol alone or combined with STI571 was effective on inhibiting the expression of CSC-associated molecules and the CSC characteristics of melanoma B16 cells.

## 5. Conclusions 

Resveratrol could suppress the *α*-MSH signaling and CSC characteristics in melanoma cells. It implicates that resveratrol may have potential to be developed as a novel therapeutic agent against CSCs of melanoma.
